# Early-Life Iron Deficiency and Subsequent Repletion Alters Development of the Colonic Microbiota in the Pig

**DOI:** 10.3389/fnut.2019.00120

**Published:** 2019-08-07

**Authors:** Laura C. Knight, Mei Wang, Sharon M. Donovan, Ryan N. Dilger

**Affiliations:** ^1^Piglet Nutrition & Cognition Laboratory, Department of Animal Sciences, University of Illinois, Urbana, IL, United States; ^2^Division of Nutritional Sciences, University of Illinois, Urbana, IL, United States; ^3^Department of Food Science and Human Nutrition, University of Illinois, Urbana, IL, United States

**Keywords:** iron deficiency, anemia, iron repletion, pediatric nutrition, comparative nutrition, volatile fatty acids, microbiota, pig

## Abstract

**Background:** Iron deficiency is the most prevalent micronutrient deficiency worldwide, affecting over two billion people. Early-life iron deficiency may alter the developing microbiota, which may or may not be reversible with subsequent dietary iron repletion. Thus, the aim of this study was to determine whether early-life iron deficiency and subsequent repletion alter colonic microbial composition and fermentation end-product concentrations in pigs.

**Methods:** Forty-two male pigs received either control (CONT, 21.3 mg Fe/L) or iron-deficient (ID, 2.72 mg Fe/L) milk replacer treatments from postnatal day (PND) 2 to 32. Subsequently, 20 pigs continued through a series of age-appropriate, iron-adequate diets from PND 33 to 61. Contents from the ascending colon (AC) and rectum (feces) were collected at PND 32 and/or 61. Assessments included microbiota composition by 16S rRNA sequencing and volatile fatty acid (VFA) concentrations by gas chromatography methods. Data were analyzed using a 1-way ANOVA and PERMANOVA to assess the main effects of early-life iron status on all outcomes.

**Results:** In AC samples, 15 genera differed (*P* < 0.05) between ID and CONT pigs, while 27 genera differed (*P* < 0.05) in fecal samples at PND 32. Early-life ID pigs had higher (*P* = 0.012) relative abundance of *Lactobacillus* in AC samples compared with CONT pigs. In feces, ID pigs had lower (*P* < 0.05) relative abundances of *Bacteroides* and *Clostridium* from the families of Clostridiaceae, Lachnospiraceae, and Ruminococcaceae. At PND 61, only two genera differed between treatment groups in AC samples, with ID pigs having a higher (*P* = 0.043) relative abundance of *Bifidobacterium* and lower (*P* = 0.047) relative abundance of *Prevotella*. Beta diversity differed at PND 32 in both AC and feces between CONT and ID pigs but no differences remained at PND 61. At PND 32, the total VFA concentration was higher in ID pigs compared with CONT pigs in both AC (*P* = 0.003) and feces (*P* = 0.001), but no differences in VFA concentrations persisted to PND 61.

**Conclusion:** Early-life iron status influenced microbial composition and VFA concentrations within the large intestine, but these differences were largely normalized following subsequent dietary iron repletion.

## Introduction

Early-life nutrition profoundly influences neonatal development, with some deficiencies leading to long-term alterations, as is the case for iron. Iron is an essential micronutrient for many biological processes, yet it is the leading micronutrient deficiency worldwide. Iron deficiency affects over two billion people, with women of childbearing age and young children being most vulnerable to deficiency (1, 2). Maternal iron deficiency can lead to reduced iron stores in the infant, who is already of heightened risk of developing iron deficiency due to rapid growth after birth (3, 4). When untreated, iron deficiency can progress to iron deficiency anemia (IDA), which has more severe effects on the developing infant.

Of particular interest are the effects of IDA on microbiota composition, which primarily develops during infancy (5). Moreover, a growing body of evidence suggests that iron availability within the gastrointestinal tract (GIT) may influence the bacterial species thriving in that environment. The microbiome is now known to have long-lasting effects on the host, including implications in autoimmune, metabolic, and gastrointestinal diseases, as well as influencing the development of allergies (5, 6). Nearly all microbial fermentation end-products, e.g., volatile fatty acids (VFA), are produced within the lumen of the large intestine (7). As such, determining how early-life iron deficiency followed by dietary iron repletion influences the composition of the neonatal microbiota is imperative to identify if iron deficiency-induced alterations are reversible.

The current study utilized the young pig as a translational model for the human infant. The pig shows similarities in nutrient requirements, and intestinal morphology and function of the GIT to the human infant (8). Further, iron deficiency is a common micronutrient deficiency in the young pig due to low levels of iron stores at birth, and for many reasons that parallel those seen in the infant, including, low iron concentration in porcine milk, heightened growth trajectories after birth, and immature iron absorption pathways until later in life (9). As such, the pig is a robust pre-clinical model for early-life iron deficiency (10–13) and a powerful model for early-life nutrition research (14). Importantly, the lean tissue accretion rate of domestic pigs far exceeds that of humans, which means that pigs will exhibit clinical signs of ID anemia by 3 weeks of age if not provided supplemental iron early in the postnatal period. This is in contrast to human infants, who are capable of relying on iron derived from body stores and that found in exogenous sources (i.e., human milk or infant formula) for approximately the first 4-to-6-months of age. It should be noted that differences in the microbiota between formula-fed and breastfed infants have been established (5, 15, 16), thus, it can be expected that differences would occur between piglets fed formula vs. porcine milk as well; this theory warrants further research.

The aims of this study were to characterize the effects of early-life IDA on development of the microbiota and to assess if subsequent dietary iron repletion would reverse the effects of early-life iron deficiency. Iron deficiency anemia was well-established in the iron deficient (ID) group during phase 1 of the current study as described elsewhere (17). To characterize these alterations, the microbiota and fermentation end-product profiles were evaluated at PND 32, and again at study conclusion (PND 61).

## Materials and Methods

### Animal Care and Use

All animal and experimental procedures were in accordance with the National Research Council Guide for the Care and Use of Laboratory Animals and approved by the University of Illinois at Urbana-Champaign Institutional Animal Care and Use Committee. Forty-two naturally-farrowed, intact (i.e., not castrated) male pigs were obtained from a commercial swine farm in two replicate groups and transferred to the University of Illinois Piglet Nutrition and Cognition Laboratory (PNCL) at PND 2. Nine pigs were omitted from tissue analysis due to failure to thrive or complications during experimental procedures that are reported elsewhere (18, 19). Per standard agricultural protocol, pigs were administered a single intramuscular injection of antibiotic (0.1 mL of ceftiofur crystalline free acid as Excede, Zoetis, Parsippany, NJ) within 24 h of birth. Contrary to typical agricultural procedures, pigs on this study were never provided supplemental iron (i.e., injectable iron dextran) due to the experimental focus on this nutrient. Recent pig studies observed hippocampal transcriptome changes (20) and possible effects of iron overload (11) after iron dextran administration in the first few days of life, which further justifies our experimental protocol. Upon arrival to PNCL on PND 2, pigs were randomized to one of two experimental milk replacer treatments (described below). Pigs were provided experimental milk replacer diets from PND 2 until PND 32 or 33 (phase 1), at which point both treatment groups were transitioned through a series of industry-standard diets from PND 32 or 33 until PND 61 or 62 (phase 2).

For phase 1 of this study, 42 piglets were housed individually in custom pig rearing units (87.6 cm long, 88.9 cm wide, 50.8 cm high), which were composed of three acrylic walls, one stainless steel wall, and vinyl-coated, expanded metal flooring. This caging environment was designed for pigs to see, hear, and smell, but not touch neighboring pigs. Pigs were allowed to physically interact with one another for approximately 15 min each day, and each pig was provided a toy for enrichment in their home-cage throughout the study. Facility lighting was maintained on a 12 h light and dark cycle starting at 0800, with ambient temperature set at 27°C for the first 21 days of the study and gradually lowered to 22°C during the last seven days of phase 1.

For phase 2 of this study, 20 pigs (*n* = 10 per diet) from phase 1 were transferred to the University of Illinois Veterinary Medicine Research Farm on PND 32 or 33 until the end of the study. While in this facility, pigs were housed individually in floor pens (1.5 m^2^) and the rearing environment remained on a 12 h light and dark cycle starting at 0800, with ambient temperature set at 22°C.

### Dietary Treatments

For phase 1 of this study, pigs (*n* = 21 per diet) were provided one of two milk replacer treatments with varying iron content. The control diet (CONT) was formulated to meet all of the nutrient requirements of the growing pig and was formulated to contain 106.3 mg Fe/kg milk replacer powder. The ID diet was identical to the CONT diet, with the exception that ferrous sulfate (i.e., the predominant iron source in CONT) was removed, such that this treatment provided only 13.6 mg Fe/kg milk replacer powder. Additionally, both diets were formulated to contain ARA and DHA at 2.08 g and 1.04 g DHA/kg milk replacer powder, respectively. Milk replacer was reconstituted fresh daily with 200 g of milk replacer powder per 800 g water. Thus, formulated iron concentrations in reconstituted pig milk replacer treatments were 21.3 and 2.72 mg Fe/L milk replacer for the CONT and ID treatments, respectively. All pigs were provided *ad libitum* access to liquid milk replacer treatments for a 20 h feeding period each day from PND 2 until PND 32 or 33.

**Figure 1:**
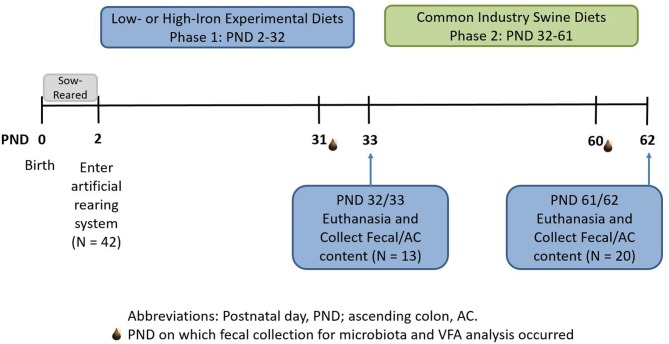
Experimental timeline. Piglets were born on postnatal day (PND) 0 and sow-reared for the first 2 days of life. On PND 2, 42 intact male piglets were brought into the artificial rearing system and placed on either an iron deficient (ID) or control (CONT) milk replacer until PND 32. Nine pigs were removed from the study due to failure to thrive or complications during neuroimaging procedures. At PND 32 (*N* = 13; CONT, *n* = 6; ID, *n* = 7), 13 pigs were euthanized to allow tissue collection. Twenty pigs from phase 1 continued on to common industry swine diets during phase 2. At PND 61 or 62 (*N* = 20; *n* = 10 per phase 1 diet), remaining pigs were euthanized to allow tissue collection at the end of the study. CONT, control diet; ID, iron deficient diet; PND, postnatal day.

**Figure 2:**
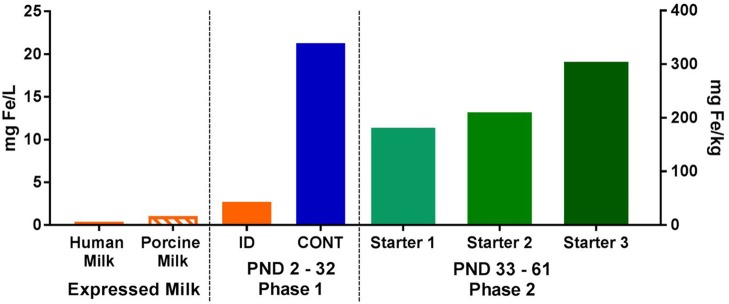
Analyzed concentrations of iron expressed human and porcine milk, as well as in dietary treatments during both phases of the pig study. During phase 1, pigs were fed either a control (CONT) or iron-deficient (ID) milk replacer. The CONT treatment contained 21.3 mg/L (106.3 mg/kg) and the ID treatment contained 2.72 mg/L (13.6 mg/kg). The ID treatment closely resembled the average iron content of porcine milk (*n* = 7; 1.06 mg/L) collected during a prior study (22), and is comparable to the iron concentration found in human milk (23, 24). During phase 2, all pigs were fed a series of nutritionally-adequate, age-appropriate diets (180-300 mg/kg). CONT, control diet; ID, iron deficient diet; PND, postnatal day.

For phase 2 of this study, all pigs were transitioned through a common series of age-appropriate, industry-relevant, iron-adequate diets (containing 180–300 mg Fe/kg of diet), regardless of their phase 1 dietary iron treatment group. Pigs were provided *ad libitum* access to standard diets (major ingredients including corn, whey, and soybean meal) and standard agricultural feeding practices were followed by sequentially switching to stage 1, 2, and 3 diets on PND 32, 41, and 50, respectively. A detailed timeline can be found in Figure 1. During phase 2 of the study, all diets were formulated to meet all nutrient requirements of growing pigs (21), including iron. No zinc oxide, copper sulfate, or in-feed antibiotics were included in any diets. Analyzed concentrations of iron in all dietary treatments can be found in Figure 2.

Porcine milk was collected as part of a previous study (22). Samples were then analyzed for mineral profiles by using standardized procedures (Mead Johnson Nutrition, Evansville, IN) to establish iron content. Specifically, porcine milk samples were digested using a combination of concentrated nitric acid and 30% hydrogen peroxide at 220°C for 10 min in a microwave digestion system (UltraWAVE; Milestone Inc., Shelton, CT). After digestion, the samples were diluted to volume and quantified by inductively-coupled plasma mass spectrometry (ICP-MS; NexION 300D; Perkin Elmer, Waltham, MA). The instrument was operated in kinetic energy discrimination mode using helium to reduce polyatomic interferences. All samples were analyzed in duplicate.

### Sample Collection, Processing, and Analysis

Fecal samples were collected utilizing a fecal loop on PND 31 (CONT, *n* = 6; ID, *n* = 7) and/or PND 60 (*N* = 20; *n* = 10 per phase 1 diet), snap frozen in liquid nitrogen, and stored at −80°C until processing. At PND 32 (CONT, *n* = 6; ID, *n* = 7) and PND 61 or 62 (*N* = 20; *n* = 10 per phase 1 diet), pigs were euthanized to allow tissue collection. All animals were euthanized in a food-deprived state, with access only to water for at least 6 h prior to euthanasia. Pigs were anesthetized using an intramuscular injection of telazol:ketamine:xylazine administered at 0.022 mL/kg bodyweight (50.0 mg tiletamine plus 50.0 mg of zolazepam reconstituted with 2.50 mL ketamine (100 g/L) and 2.50 mL xylazine (100 g/L); Fort Dodge Animal Health, Overland Park, KS). Pigs were euthanized using a 390 mg/mL sodium pentobarbital solution (Patterson Veterinary Supply, Columbus, OH) at 1 mL/5 kg body weight with an intracardiac injection. On the day of tissue collection, AC and fecal samples were collected after euthanasia at PND 32 or PND 61, snap frozen in liquid nitrogen, and stored at −80°C until processing.

### Microbiota Analysis

#### DNA Extraction

DNA was extracted from AC contents and feces utilizing the QIAamp DNA Stool Mini Kit (Qiagen, Valencia, CA) in combination with the FastPrep-24 System (MP Biomedicals, Carlsbad, CA), as previously described (25). DNA quality was assessed on a 1% agarose gel following staining with ethidium bromide, and DNA concentrations were quantified using a NanoDrop 1000 spectrophotometer (NanoDrop Technologies, Wilmington, DE).

#### PCR Amplification and Sequencing of 16S rRNA Genes

PCR amplification and sequencing of V3-V4 regions of bacterial 16S rRNA genes were performed at the DNA services Lab, University of Illinois, as previously described (26).

#### Sequence Processing

Sequences were demultiplexed at the sequencing facility with the bcl2fastq v2.17.1.14 Conversion Software (Illumina, San Diego, CA), allowed 0 mismatches in the barcode sequences. De-multiplexed forward (read 1) and reverse reads (read 2) were further processed using the QIIME software package (27) The paired-end reads were merged, filtered and split into libraries as previously described (26). The representative operational taxonomic units (OTU) picking, chimera removing and construction of phylogenetic tree were performed as described by Monaco et al. (26). The representative sequence of each OTU was assigned to different taxonomic levels using Ribosomal Database Project naïve Bayesian rRNA Classifier (28) at 80% confidence level on the Greengenes reference database v.13.8. An OTU table was created and further filtered to remove non-aligned and chimeric OTUs and singletons. Alpha diversity (observed OTUs, Chao1 and Shannon and Simpson reciprocal indices) and beta diversity analysis was performed from the filtered OTU table after rarefying to 26,350 reads for each sample.

#### Volatile Fatty Acid Analysis

Both AC and fecal samples from phase 1 and phase 2 were utilized to quantify VFA concentrations. As such, AC samples were thawed, weighed (100 mg each), and acidified with an equal volume of 2 N HCl. Fecal samples were thawed, weighed (100 mg each), and acidified using 6.25% m-phosphoric acid, sonicated, and stored overnight at −20°C. Samples were then thawed and centrifuged for 10 min at 16,500 × g, and the supernatant was collected for analysis via gas chromatography. All samples were assessed as previously described (29). Acetic, n-butyric, propionic, valeric, isovaleric, and isobutyric acid solutions were used as standards (Sigma Aldrich, St. Louis, MO) to quantify individual VFA concentrations.

#### Statistical Analysis

All researchers involved in this study (i.e., those performing daily procedures, data collection, and data analysis steps) remained blinded to dietary treatment identity until final data analyses had been completed. Differences in bacterial communities among diet groups were evaluated with principal co-ordinate analysis (PCoA) and permutational multivariate analysis of variance (PERMANOVA) using UniFrac distance matrices (30). PCoA and PERMANOVA were performed on both unweighted and weighted UniFrac distances using QIIME (27). All other data were analyzed using the MIXED procedure of SAS (version 9.4, SAS Institute, Cary, NC). Replicate was considered a random variable. All data were collected at an individual time-point, and thus were analyzed using a one-way ANOVA to determine the effect of phase 1 dietary iron status. Relative abundances of all phyla and genera of 0.05% or greater were analyzed and were arcsin transformed before analysis. Outliers, defined as having a studentized residual with an absolute value > 3, were removed from the dataset prior to statistical analysis. Significance was accepted at *P* ≤ 0.05, and data are presented as least-squares means with pooled standard errors of the mean (SEM). For relative abundance microbiota outcomes, data are presented as raw least-square means plus SEM, along with arcsin-transformed *P*-values.

## Results

### Microbiota

#### Ascending Colon

**Figure 3:**
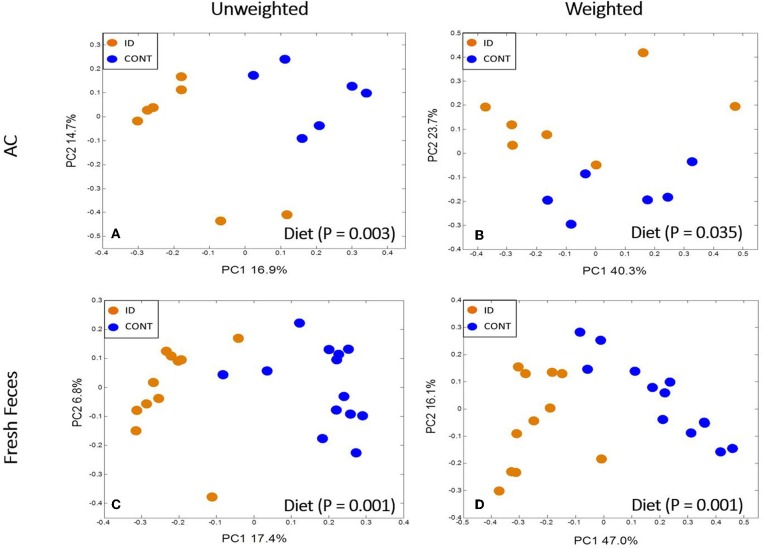
Principal co-ordinate analysis of unweighted and weighted UniFrac distances generated from microbiota of AC and fecal samples of piglets fed different diets at PND 32. **(A)** Unweighted; Generated from ascending colon contents of piglets fed CONT (*n* = 6) or ID (*n* = 7) diets. Adonis *P* = 0.003. **(B)** Weighted; Generated from ascending colon contents of piglets fed CONT (*n* = 6) or ID (*n* = 7) diets. Adonis *P* = 0.035. **(C)** Unweighted; Generated from feces of piglets fed CONT (*n* = 13) or ID (*n* = 11) diets. Adonis *P* = 0.001. **(D)** Weighted; Generated from feces of piglets fed CONT (*n* = 13) or ID (*n* = 11) diets. Adonis *P* = 0.001. CONT, control diet; ID, iron deficient diet.

Principal co-ordinate analysis of UniFrac distances produced from AC samples at PND 32 are shown in Figures 3A,B. PERMANOVA analysis revealed that overall bacterial composition in AC samples differed (unweighted, *P* = 0.003, weighted *P* = 0.035) between CONT and ID pigs. At the phylum level, no differences were observed in AC (Table 1). At the genus level, CONT pigs displayed higher (*P* ≤ 0.034) relative abundances of *Akkermansia, Anaerotruncus, Barnesiella, Bilophila, Butyricimonas, Collinsella, Eggerthella, Parabacteroides, [Ruminococcus]* (taxa names with brackets indicate proposed taxonomies that Greengenes recommends based on the whole genome phylogeny, but they are not officially recognized by Bergey's manual of systematic bacteriology), and *Sutterella*, and ID pigs displayed higher (*P* ≤ 0.020) levels of *Dialister, Lactobacillus, Megasphaera, Prevotella*, and *[Prevotella]* in AC samples (Table 2). In AC samples, no differences were observed in Shannon (*P* = 0.761) or Chao 1 (*P* = 0.594) indices, or in the Simpson reciprocal index (*P* = 0.568) at PND 32.

**Table 1:** Relative abundances of bacterial phyla detected in ascending colon and fecal samples at PND32^a^.

	**Ascending Colon**		**Feces**	
**Phyla**	**CONT, *n* = 6**	**ID, *n* = 7**	**CONT, *n* = 13**	**ID, *n* = 11**
Actinobacteria	0.24 ± 0.46	0.71 ± 0.44	0.71 ± 0.87	3.72 ± 0.94^*^
Bacteroidetes	62.4 ± 5.05	56.3 ± 4.67	45.3 ± 3.17	43.9 ± 3.45
Cyanobacteria	0.02 ± 0.02	0.02 ± 0.02	0.08 ± 0.05	0.05 ± 0.05
Deferribacteres	0.00 ± 0.01	0.00 ± 0.01	0.00 ± 0.00	0.00 ± 0.00
Firmicutes	23.9 ± 7.7	25.5 ± 7.55	44.0 ± 3.53	41.2 ± 3.84
Fusobacteria	5.06 ± 2.27	4.77 ± 2.24	1.32 ± 1.43	1.90 ± 1.45
Lentisphaerae	0.01 ± 0.01	0.02 ± 0.01	0.00 ± 0.01	0.02 ± 0.01
Planctomycetes	0.00 ± 0.00	0.00 ± 0.00	0.01 ± 0.01	0.01 ± 0.01
Proteobacteria	5.26 ± 3.53	10.2 ± 3.48	3.19 ± 0.96	6.80 ± 1.05^*^
Spirochaetes	0.00 ± 0.01	0.02 ± 0.01	0.00 ± 0.01	0.02 ± 0.01
Synergistetes	0.34 ± 0.15	0.06 ± 0.14	0.47 ± 0.16	0.34 ± 0.17
Tenericutes	0.20 ± 0.13	0.13 ± 0.13	0.09 ± 0.10	0.28 ± 0.11
Verrucomicrobia	0.07 ± 0.03	0.00 ± 0.03	1.33 ± 0.51	0.06 ± 0.55
WPS-2	0.31 ± 0.20	0.07 ± 0.20	0.16 ± 0.15	0.11 ± 0.15
Unknown bacteria	1.78 ± 0.34	1.93 ± 0.34	1.61 ± 0.60	2.17 ± 0.61^*^

a *Data are expressed as mean ± standard error of the mean (SEM) for each dietary treatment group. Main effects of dietary treatment (Diet; CONT vs. ID) are presented. CONT, control diet; ID, iron deficient diet; PND, postnatal day; SEM, standard error of the mean*.

* *Within same intestinal segment and row, CONT and ID groups differ, P ≥ 0.05*.

**Table 2:** Relative abundances of bacterial genera detected in ascending colon contents at PND32^a^.

**Bacterial genus^b^**	**CONT, *n* = 6**	**ID, *n* = 7**	***P*-value*^d^***
**Actinobacteria**			
*Collinsella*	0.05 ± 0.02	0.00 ± 0.02	0.036^*^
*Eggerthella*	0.05 ± 0.02	0.00 ± 0.02	0.006^*^
**Bacteroidetes**			
*Alistipes*	0.77 ± 0.19	0.29 ± 0.18	0.057
*Bacteroides*	27.91 ± 6.96	15.49 ± 6.45	0.209
*Barnesiella*	0.13 ± 0.05	0.01 ± 0.05	0.002^*^
*Butyricimonas*	3.74 ± 1.37	1.00 ± 1.32	0.021^*^
*Odoribacter*	4.08 ± 2.87	1.77 ± 2.86	0.074
*Parabacteroides*	11.87 ± 5.66	3.52 ± 5.58	0.042^*^
*Prevotella*	1.48 ± 4.65	7.14 ± 4.58	0.010^*^
*[Prevotella]^c^*	1.83 ± 2.77	11.37 ± 2.65	0.020^*^
**Firmicutes**			
*Acidaminococcus*	0.00 ± 0.70	0.90 ± 0.64	0.230
*Anaerofilum*	0.05 ± 0.02	0.01 ± 0.02	0.073
*Anaerotruncus*	0.10 ± 0.05	0.03 ± 0.05	0.034^*^
*Anaerovibrio*	0.08 ± 0.06	0.01 ± 0.06	0.161
*Blautia*	1.33 ± 0.94	1.05 ± 0.91	0.632
*Christensenella*	0.07 ± 0.03	0.01 ± 0.03	0.069
*Clostridium* (Clostridiaceae)	0.32 ± 0.32	0.56 ± 0.31	0.548
*Clostridium* (Lachnospiraceae)	0.66 ± 0.20	0.18 ± 0.19	0.058
*Clostridium* (Ruminococcaceae)	0.09 ± 0.05	0.01 ± 0.04	0.160
*Coprococcus*	0.10 ± 0.04	0.08 ± 0.03	0.759
*Dialister*	0.00 ± 0.02	0.07 ± 0.01	0.001^*^
*Dorea*	1.39 ± 0.66	1.05 ± 0.62	0.642
*Epulopiscium*	0.39 ± 2.13	2.54 ± 2.06	0.297
*[Eubacterium]*	0.51 ± 0.22	0.17 ± 0.21	0.190
*Faecalibacterium*	0.53 ± 0.57	1.50 ± 0.54	0.190
*Lactobacillus*	0.31 ± 2.25	5.99 ± 2.11	0.012^*^
*Lactococcus*	0.29 ± 0.10	0.09 ± 0.10	0.354
*Leuconostoc*	0.34 ± 0.16	0.06 ± 0.15	0.413
*Megaspheara*	0.00 ± 0.07	0.14 ± 0.06	0.015^*^
*Mitsuokella*	0.00 ± 0.23	0.32 ± 0.21	0.128
*Oscillospira*	2.20 ± 1.11	1.70 ± 1.08	0.519
*Phascolarctobacterium*	0.16 ± 0.08	0.02 ± 0.08	0.150
*p-75-a5*	0.14 ± 0.18	0.21 ± 0.18	0.765
*Ruminococcus*	0.14 ± 0.03	0.10 ± 0.03	0.304
*[Ruminococcus]^c^*	2.91 ± 0.49	0.79 ± 0.46	0.003^*^
*RFN20*	0.00 ± 0.18	0.35 ± 0.16	0.134
**Fusobacteria**			
*Fusobacterium*	3.17 ± 1.21	2.23 ± 1.16	0.521
**Proteobacteria**			
*Bilophila*	0.14 ± 0.06	0.02 ± 0.06	0.019^*^
*Campylobacter*	0.04 ± 1.12	2.31 ± 1.04	0.082
*Desulfovibrio*	1.15 ± 0.34	0.38 ± 0.31	0.101
*Escherichia*	0.75 ± 1.41	2.48 ± 1.36	0.199
*Flexispira*	0.03 ± 0.11	0.16 ± 0.11	0.068
*Helicobacter*	0.11 ± 0.38	0.54 ± 0.37	0.051
*Pasteurella*	0.07 ± 0.05	0.02 ± 0.05	0.977
*Sutterella*	1.53 ± 0.32	0.20 ± 0.29	0.011^*^
**Synergistetes**			
*Synergistes*	0.25 ± 0.11	0.04 ± 0.10	0.173
**Verrucomicrobia**			
*Akkermansia*	0.07 ± 0.03	0.00 ± 0.03	0.006^*^

a *Data are expressed as mean ± standard error of the mean (SEM) for each dietary treatment group. Main effects of dietary treatment (Diet; CONT vs. ID) are presented. CONT, control diet; ID, iron deficient diet; PND, postnatal day; SEM, standard error of the mean*.

b *Only bacterial genera with mean relative abundance >0.05% were analyzed. Data are expressed as mean ± SEM*.

c *Taxa that have brackets around the names are proposed taxonomies that Greengenes recommends based on the whole genome phylogeny but not officially recognized by Bergey's manual of systematic bacteriology*.

d *P-values for the main effect of early-life dietary iron status, which were arcsin transformed before analysis*.

* *CONT and ID groups differ, P ≤ 0.05*.

**Figure 4:**
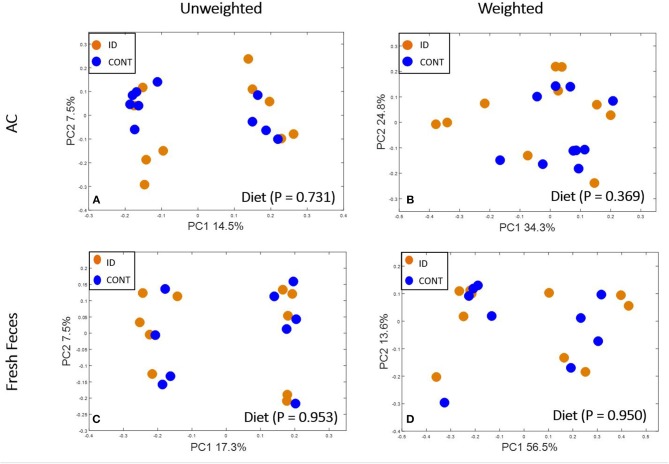
Principal co-ordinate analysis of unweighted and weighted UniFrac distances generated from microbiota of AC and fecal samples of piglets fed different diets at PND 61. **(A)** Unweighted; Generated from ascending colon contents of piglets fed CONT (*n* = 10) or ID (*n* = 10) diets. Adonis *P* = 0.731. **(B)** Weighted; Generated from ascending colon contents of piglets fed CONT (*n* = 10) or ID (*n* = 10) diets. Adonis *P* = 0.369. **(C)** Unweighted; Generated from feces of piglets fed CONT (*n* = 9) or ID (*n* = 10) diets. Adonis *P* = 0.953. **(D)** Weighted; Generated from feces of piglets fed CONT (*n* = 9) or ID (*n* = 10) diets. Adonis *P* = 0.950. CONT, control diet; ID, iron deficient diet.

At PND 61, PCoA of UniFrac distances produced from AC samples are shown in Figures 4A,B. PERMANOVA analysis revealed that overall bacterial composition in AC samples at PND 61 did not differ (unweighted, *P* = 0.731, weighted *P* = 0.369) between treatment groups. No differences were observed in AC at the phylum level (Table 3). At the genus level, CONT pigs displayed higher (*P* = 0.047) relative abundance of *Prevotella*, and ID pigs had increased (*P* = 0.043) relative abundance of *Bifidobacterium* and trending higher (*P* = 0.095) *Megashpeara* in AC samples at PND 61 (Table 4). No differences were observed in Shannon (*P* = 0.128) or Chao 1 (*P* = 0.153) indices, or the Simpson reciprocal index (*P* = 0.118) in AC samples at PND 61.

**Table 3:** Relative abundances of bacterial phyla detected in ascending colon and fecal samples at PND61^a^.

	**Ascending Colon**		**Feces**	
**Phyla**	**CONT, *n* = 10**	**ID, *n* = 10**	**CONT, *n* = 9**	**ID, *n* = 10**
Actinobacteria	0.07 ± 0.03	0.15 ± 0.03	0.34 ± 0.10	0.21 ± 0.10
Bacteroidetes	59.05 ± 2.14	56.71 ± 2.14	47.73 ± 6.79	45.70 ± 6.75
Cyanobacteria	2.04 ± 1.35	1.85 ± 1.35	0.41 ± 0.17	0.45 ± 0.16
Deferribacteres	0.01 ± 0.02	0.03 ± 0.02	0.00 ± 0.01	0.02 ± 0.01
Firmicutes	28.66 ± 2.16	32.65 ± 2.16	42.65 ± 7.82	43.94 ± 7.78
Fusobacteria	0.27 ± 0.19	0.00 ± 0.19	0.01 ± 0.00	0.00 ± 0.00
Lentisphaerae	0.01 ± 0.00	0.01 ± 0.00	0.01 ± 0.01	0.03 ± 0.01
Planctomycetes	0.02 ± 0.02	0.01 ± 0.02	0.04 ± 0.03	0.02 ± 0.03
Proteobacteria	9.05 ± 1.50	7.66 ± 1.50	4.52 ± 1.42	5.07 ± 1.39
Spirochaetes	0.07 ± 0.03	0.11 ± 0.03	1.80 ± 1.42	1.87 ± 1.41
Synergistetes	0.01 ± 0.01	0.01 ± 0.01	0.29 ± 0.15	0.27 ± 0.15
Tenericutes	0.01 ± 0.01	0.01 ± 0.01	0.12 ± 0.07	0.02 ± 0.06
TM7	0.00 ± 0.00	0.00 ± 0.00	−	−
Verrucomicrobia	0.00 ± 0.00	0.00 ± 0.00	0.00 ± 0.00	0.00 ± 0.00
WPS-2	0.27 ± 0.27	0.24 ± 0.27	0.97 ± 1.16	1.22 ± 1.15
Unknown bacteria	0.49 ± 0.03	0.53 ± 0.03	0.89 ± 0.09	1.06 ± 0.08

a *Data are expressed as mean ± standard error of the mean (SEM) for each dietary treatment group. Main effects of dietary treatment (Diet; CONT vs. ID) is presented. CONT, control diet; ID, iron deficient diet; PND, postnatal day; SEM, standard error of the mean*.

**Table 4:** Relative abundances of bacterial genera detected in ascending colon contents at PND61^a^.

**Bacterial genus^b^**	**CONT, *n* = 10**	**ID, *n* = 10**	***P*-value*^d^***
**Actinobacteria**			
*Bifidobacterium*	0.03 ± 0.03	0.11 ± 0.03	0.043^*^
**Bacteroidetes**			
*Bacteroides*	0.84 ± 0.51	1.33 ± 0.51	0.611
*Butyricimonas*	0.14 ± 0.08	0.22 ± 0.08	0.124
*Parabacteroides*	0.53 ± 0.74	0.99 ± 0.74	0.246
*Prevotella*	44.88 ± 2.93	36.03 ± 2.93	0.047^*^
*[Prevotella]^c^*	5.90 ± 1.88	8.01 ± 1.88	0.219
**Firmicutes**			
*Acidaminococcus*	0.23 ± 0.04	0.16 ± 0.04	0.281
*Anaerovibrio*	5.48 ± 1.12	4.32 ± 1.12	0.278
*Blautia*	0.60 ± 0.17	0.76 ± 0.17	0.283
*Butyricicoccus*	0.07 ± 0.02	0.05 ± 0.02	0.555
*Clostridium* (Clostridiaceae)	0.19 ± 0.15	0.25 ± 0.15	0.704
*Coprococcus*	1.40 ± 0.70	0.52 ± 0.70	0.502
*Dorea*	0.33 ± 0.14	0.36 ± 0.14	0.683
*Epulopiscium*	0.07 ± 0.09	0.10 ± 0.09	0.976
*Faecalibacterium*	0.59 ± 0.28	0.90 ± 0.28	0.472
*Lachnospira*	0.91 ± 0.59	1.18 ± 0.59	0.370
*Lactobacillus*	1.26 ± 0.59	1.80 ± 0.59	0.648
*Megaspheara*	0.67 ± 0.21	1.13 ± 0.21	0.095
*Mitsuokella*	0.62 ± 0.43	1.04 ± 0.43	0.570
*Oribacterium*	0.13 ± 0.13	0.12 ± 0.13	0.988
*Oscillospira*	1.36 ± 0.37	1.32 ± 0.37	0.750
*Phascolarctobacterium*	0.09 ± 0.02	0.10 ± 0.02	0.721
*Roseburia*	1.13 ± 0.26	1.38 ± 0.26	0.505
*Ruminococcus*	0.44 ± 0.08	0.54 ± 0.08	0.348
*[Ruminococcus]*	0.09 ± 0.02	0.08 ± 0.02	0.803
*RFN20*	0.17 ± 0.05	0.16 ± 0.05	0.993
*Sharpea*	0.02 ± 0.03	0.06 ± 0.03	0.260
*Streptococcus*	0.32 ± 0.07	0.31 ± 0.07	0.941
*Turicibacter*	0.05 ± 0.02	0.04 ± 0.02	0.536
**Proteobacteria**			
*Actinobacillus*	0.01 ± 0.05	0.06 ± 0.05	0.567
*Campylobacter*	4.31 ± 3.39	3.59 ± 3.38	0.354
*Desulfovibrio*	0.11 ± 0.09	0.16 ± 0.09	0.395
*Flexispira*	0.05 ± 0.06	0.10 ± 0.06	0.390
*Helicobacter*	0.08 ± 0.03	0.09 ± 0.03	0.585
*Sutterella*	0.25 ± 0.06	0.18 ± 0.06	0.275
**Spirochaetes**			
*Sphaerochaeta*	0.04 ± 0.03	0.06 ± 0.03	0.155
*Treponema*	0.03 ± 0.04	0.05 ± 0.04	0.591

a *Data are expressed as mean ± standard error of the mean (SEM) for each dietary treatment group. Main effects of dietary treatment (Diet; CONT vs. ID) is presented. CONT, control diet; ID, iron deficient diet; PND, postnatal day; SEM, standard error of the mean*.

b *Only bacterial genera with mean relative abundance >0.05% were analyzed. Data are expressed as mean ± SEM*.

c *Taxa that have brackets around the names are proposed taxonomies that Greengenes recommends based on the whole genome phylogeny but not officially recognized by Bergey's manual of systematic bacteriology*.

d *P-values for the main effect of early-life dietary iron status, which were arcsin transformed before analysis*.

* *CONT and ID groups differ, P ≤ 0.05*.

#### Feces

Principal co-ordinate analysis of UniFrac distances generated from fecal samples at PND 32 are shown in Figures 3C,D. PERMANOVA analysis revealed that overall bacterial composition in feces (unweighted and weighted, *P* = 0.001) differed between CONT and ID pigs. At the phylum level, ID pigs had increased relative abundances in Actinobacteria (*P* = 0.0113) and Proteobacteria (*P* = 0.019) in feces when compared with CONT pigs (Table 1). At the genus levels, CONT pigs harbored greater (*P* ≤ 0.045) proportions of *Akkermansia, Bacteroides, Barnsiella, Chrstensenella, Clostridium* from the families of *Clostridiaceae, Lachnospiraceae*, and *Ruminococcaceae, Eggerthella, [Eubacterium], [Ruminococcus]*, and *Rothia*, and ID pigs had increased (*P* ≤ 0.041) abundance in *Acidaminococcus, Bifidobacterium, Bilophila, Coprococcus, Dialister, Escherichia, Faecalibacterium, Lactobacillus, Megasphaera, Mitsuikella, Oscilospira, Prevotella, [Prevotella], RFN20, Sharpea*, and *Shigella* in feces (Table 5). Iron deficient pigs had higher (*P* = 0.016) Shannon index.

**Table 5:** Relative abundances of bacterial genera detected in feces at PND32^a^.

**Bacterial genus^b^**	**CONT, *n* = 13**	**ID, *n* = 11**	***P*-value^d^**
**Actinobacteria**			
*Bifidobacterium*	0.01 ± 0.39	0.96 ± 0.43	0.041^*^
*Collinsella*	0.05 ± 0.01	0.04 ± 0.01	0.490
*Eggerthella*	0.19 ± 0.08	0.01 ± 0.09	0.029^*^
*Rothia*	0.07 ± 0.02	0.01 ± 0.02	0.011^*^
**Bacteroidetes**			
*Alistipes*	0.53 ± 0.23	0.97 ± 0.25	0.244
*Bacteroides*	30.00 ± 7.17	1.06 ± 7.52	<0.001^*^
*Barnesiella*	0.28 ± 0.22	0.18 ± 0.22	0.024^*^
*Butyricimonas*	1.29 ± 0.52	1.46 ± 0.55	0.883
*Odoribacter*	2.13 ± 2.28	5.20 ± 2.42	0.193
*Parabacteroides*	2.51 ± 1.41	2.22 ± 1.43	0.520
*Prevotella*	0.90 ± 1.46	2.78 ± 1.49	<0.001^*^
*[Prevotella]^c^*	0.20 ± 1.84	9.99 ± 2.00	<0.001^*^
**Firmicutes**			
*Acidaminococcus*	0.00 ± 0.07	0.26 ± 0.07	<0.001^*^
*Anaerofilum*	0.04 ± 0.01	0.02 ± 0.01	0.053
*Anaerotruncus*	0.16 ± 0.04	0.13 ± 0.04	0.703
*Blautia*	1.67 ± 0.84	3.44 ± 0.91	0.189
*Christensenella*	0.18 ± 0.05	0.03 ± 0.06	0.001^*^
*Clostridium* (Clostridiaceae)	2.44 ± 0.79	0.17 ± 0.86	0.010^*^
*Clostridium* (Lachnospiraceae)	1.18 ± 0.37	0.55 ± 0.39	0.013^*^
*Clostridium* (Peptostreptococcaceae)	0.09 ± 0.03	0.02 ± 0.03	0.053
*Clostridium* (Ruminococcaceae)	0.06 ± 0.02	0.01 ± 0.02	0.011^*^
*Coprococcus*	0.10 ± 0.06	0.18 ± 0.06	0.017^*^
*Dialister*	0.00 ± 0.02	0.11 ± 0.02	<0.001^*^
*Dorea*	1.57 ± 0.56	1.57 ± 0.61	0.717
*Enterococcus*	0.08 ± 0.06	0.01 ± 0.06	0.341
*Epulopiscium*	1.46 ± 2.33	2.93 ± 2.37	0.134
*[Eubacterium]*	3.18 ± 1.15	0.06 ± 1.24	0.008^*^
*Faecalibacterium*	0.37 ± 0.28	1.48 ± 0.30	0.005^*^
*Lactobacillus*	1.10 ± 0.78	4.12 ± 0.85	0.001^*^
*Lactococcus*	0.07 ± 0.10	0.20 ± 0.11	0.633
*Leuconostoc*	1.05 ± 0.49	0.04 ± 0.53	0.158
*Megasphaera*	0.00 ± 0.05	0.29 ± 0.05	<0.001^*^
*Mitsuokella*	0.00 ± 0.05	0.23 ± 0.06	<0.001^*^
*Mogibacterium*	0.04 ± 0.01	0.05 ± 0.01	0.124
*Oscillospira*	2.25 ± 0.81	5.47 ± 0.88	0.006^*^
*Phascolarctobacterium*	0.07 ± 0.02	0.03 ± 0.02	0.485
*p-75-a5*	0.40 ± 0.17	0.03 ± 0.19	0.235
*Ruminococcus*	0.23 ± 0.06	0.29 ± 0.06	0.602
*[Ruminococcus]^c^*	3.46 ± 0.52	1.77 ± 0.56	0.045^*^
*RFN20*	0.00 ± 0.14	0.33 ± 0.15	0.039^*^
*Sharpea*	0.00 ± 0.09	0.31 ± 0.10	0.003^*^
*Streptococcus*	0.07 ± 0.03	0.01 ± 0.03	0.151
**Fusobacteria**			
*Fusobacterium*	0.09 ± 0.03	0.04 ± 0.04	0.209
**Proteobacteria**			
*Bilophila*	0.03 ± 0.02	0.10 ± 0.02	0.037^*^
*Campylobacter*	0.09 ± 0.14	0.27 ± 0.15	0.527
*Desulfovibrio*	1.68 ± 0.29	1.07 ± 0.32	0.130
*Escherichia*	0.87 ± 0.99	4.62 ± 1.07	0.009^*^
*Shigella*	0.01 ± 0.01	0.05 ± 0.01	0.004^*^
*Sutterella*	0.12 ± 0.03	0.04 ± 0.04	0.197
**Synergistetes**			
*Synergistes*	0.35 ± 0.12	0.24 ± 0.13	0.753
**Verrucomicrobia**			
*Akkermansia*	0.13 ± 0.50	0.06 ± 0.54	0.010^*^

a *Data are expressed as mean ± standard error of the mean (SEM) for each dietary treatment group. Main effects of dietary treatment (Diet; CONT vs. ID) are presented. CONT, control diet; ID, iron deficient diet; PND, postnatal day; SEM, standard error of the mean*.

b *Only bacterial genera with mean relative abundance >0.05% were analyzed. Data are expressed as mean ± SEM*.

c *Taxa that have brackets around the names are proposed taxonomies that Greengenes recommends based on the whole genome phylogeny but not officially recognized by Bergey's manual of systematic bacteriology*.

d *P-values for the main effect of early-life dietary iron status, which were arcsin transformed before analysis*.

* *CONT and ID groups differ, P ≤ 0.05*.

Principal co-ordinate analysis of UniFrac distances produced from fecal samples at PND 61 are shown in Figures 4C,D. PERMANOVA analysis revealed that overall bacterial composition in fecal samples at PND 61 did not differ (unweighted, *P* = 0.953, weighted *P* = 0.950) between CONT and ID pigs. At the phyla level, no differences were observed in feces at PND 61 (Table 3). At the genus level, CONT pigs displayed higher (*P* = 0.009) relative abundance of *Methanobrevibacter* in feces at study conclusion (Table 6). No differences were observed in alpha diversity between ID and CONT pigs at PND 61 in the Shannon index (*P* = 0.752), Chao 1 index (*P* = 0.502), or Simpson reciprocal (*P* = 0.925). Alpha diversity due to early-life iron deficiency at PND 32 and 61 are presented in Tables 7, 8, respectively.

**Table 6:** Relative abundances of bacterial genera detected in feces at PND61^a^.

**Bacterial genus^b^**	**CONT, *n* = 9**	**ID, *n* = 10**	***P*-value^d^**
**Actinobacteria**			
*Bifidobacteria*	0.21 ± 0.05	0.11 ± 0.05	0.223
**Bacteroidetes**			
*Alistipes*	0.05 ± 0.02	0.05 ± 0.02	0.741
*Bacteroides*	5.35 ± 2.80	5.70 ± 2.78	0.743
*Butyricimonas*	0.59 ± 0.09	0.52 ± 0.09	0.665
*Parabacteroides*	2.06 ± 1.58	1.60 ± 1.57	0.675
*Prevotella*	21.67 ± 11.69	18.95 ± 11.67	0.302
*[Prevotella]^c^*	2.16 ± 0.63	1.98 ± 0.61	0.566
**Euryarchaeota**			
*Methanobrevibacter*	0.17 ± 0.08	0.09 ± 0.08	0.009^*^
**Firmicutes**			
*Acidaminococcus*	0.96 ± 0.25	0.62 ± 0.25	0.236
*Anaerovibrio*	4.54 ± 1.02	3.12 ± 1.00	0.167
*Blautia*	1.09 ± 0.40	1.11 ± 0.39	0.872
*Butyricicoccus*	0.19 ± 0.09	0.10 ± 0.09	0.131
*Clostridium* (Clostridiaceae)	0.11 ± 0.02	0.08 ± 0.02	0.399
*Coprococcus*	0.61 ± 0.41	0.53 ± 0.40	0.534
*Dorea*	0.54 ± 0.12	0.49 ± 0.12	0.792
*Faecalibacterium*	1.03 ± 0.51	1.32 ± 0.48	0.682
*Lachnospira*	0.84 ± 0.68	1.07 ± 0.68	0.525
*Lactobacillus*	1.46 ± 0.79	1.89 ± 0.77	0.797
*Megasphaera*	2.43 ± 0.56	2.93 ± 0.53	0.576
*Mitsuokella*	1.56 ± 0.78	2.00 ± 0.75	0.764
*Oribacterium*	0.09 ± 0.12	0.14 ± 0.12	0.253
*Oscillospira*	1.84 ± 0.37	2.15 ± 0.35	0.537
*Phascolarctobacterium*	0.08 ± 0.01	0.08 ± 0.01	0.968
*Roseburia*	1.08 ± 0.55	1.27 ± 0.54	0.559
*Ruminococcus*	1.09 ± 0.41	1.25 ± 0.40	0.677
*[Ruminococcus]*	0.13 ± 0.06	0.17 ± 0.06	0.821
*RFN20*	0.72 ± 0.25	0.78 ± 0.23	0.629
*Sharpea*	0.05 ± 0.03	0.09 ± 0.03	0.477
*Streptococcus*	0.82 ± 0.34	0.75 ± 0.34	0.895
**Proteobacteria**			
*Campylobacter*	0.82 ± 0.64	0.67 ± 0.62	0.687
*Desulfovibrio*	0.37 ± 0.11	0.22 ± 0.10	0.258
*Oxalobacter*	0.05 ± 0.01	0.04 ± 0.01	0.152
*Sutterella*	0.16 ± 0.11	0.21 ± 0.10	0.434
**Spirochaetes**			
*Sphaerochaeta*	0.19 ± 0.22	0.35 ± 0.22	0.142
*Treponema*	1.61 ± 1.62	1.51 ± 1.60	0.701
**Synergistetes**			
*Synergistes*	0.25 ± 0.13	0.21 ± 0.12	0.481

a *Data are expressed as mean ± standard error of the mean (SEM) for each dietary treatment group. Main effects of dietary treatment (Diet; CONT vs. ID) is presented. CONT, control diet; ID, iron deficient diet; PND, postnatal day; SEM, standard error of the mean*.

b *Only bacterial genera with mean relative abundance >0.05% were analyzed. Data are expressed as mean ± SEM*.

c *Taxa that have brackets around the names are proposed taxonomies that Greengenes recommends based on the whole genome phylogeny but not officially recognized by Bergey's manual of systematic bacteriology*.

d *P-values for the main effect of early-life dietary iron status, which were arcsin transformed before analysis*.

* *CONT and ID groups differ, P ≤ 0.05*.

**Table 7:** Within sample bacterial diversity in ascending colon content and feces at PND32^a^.

	**Ascending colon**		**Feces**	
**Alpha Diversity**	**CONT, *n* = 6**	**ID, *n* = 7**	**CONT, *n* = 13**	**ID, *n* = 11**
Observed OTU	3161 ± 678.8	3035 ± 674.5	3120 ± 177.6	3739 ± 193.0^*^
Shannon Index	7.46 ± 0.743	7.36 ± 0.739	7.22 ± 0.449	8.27 ± 0.474^*^
Simpson Reciprocal	34.69 ± 14.875	27.65 ± 14.583	35.38 ± 14.024	48.720 ± 14.328
Chao 1	6015 ± 1469.7	5677 ± 1462.4	6214 ± 395.0	6955 ± 429.4

a *Data are expressed as mean ± standard error of the mean (SEM) for each dietary treatment group. Main effects of dietary treatment (Diet; CONT vs. ID) are presented. CONT, control diet; ID, iron deficient diet; PND, postnatal day; SEM, standard error of the mean; OTU, operational taxonomic unit*.

* *Within same intestinal segment and row, CONT and ID groups differ, P ≤ 0.05*.

**Table 8:** Within sample bacterial diversity in ascending colon content and feces at PND61^a^.

	**Ascending Colon**		**Feces**	
**Alpha Diversity**	**CONT, *n* = 10**	**ID, *n* = 9**	**CONT, *n* = 9**	**ID, *n* = 10**
Observed OTU	3103 ± 112.9	3356 ± 112.9	4006 ± 437.3	3915 ± 435.4
Shannon Index	7.16 ± 0.166	7.54 ± 0.166	7.99 ± 0.562	8.06 ± 0.559
Simpson Reciprocal	22.93 ± 4.0770	30.36 ± 4.0629	45.03 ± 21.75	44.15 ± 21.64
Chao 1	6344 ± 245.8	6864 ± 245.8	8075 ± 858.6	7777 ± 852.5

a *Data are expressed as mean ± standard error of the mean (SEM) for each dietary treatment group. Main effects of dietary treatment (Diet; CONT vs. ID) is presented. CONT, control diet; ID, iron deficient diet; PND, postnatal day; SEM, standard error of the mean; OTU, operational taxonomic unit*.

### Dry Matter and Volatile Fatty Acid Composition

#### Ascending Colon

At PND 32, ID pigs had decreased (*P* = 0.006) percent dry matter (DM) in AC contents, and the total VFA concentration in AC contents from ID pigs was increased (*P* = 0.003) compared with CONT pigs (Table 9). Absolute concentrations of acetate (*P* = 0.002), propionate (*P* = 0.018), butyrate (*P* = 0.012), and valerate (*P* = 0.025) were more than doubled in AC contents of ID pigs compared with CONT pigs. Relative to total VFA (i.e., % total VFA) concentrations, acetate was increased (*P* < 0.001) in ID pigs, but a decrease (*P* < 0.001) in relative propionate was observed in AC contents. Iron deficient pigs also had lower relative proportions of isobutyrate (*P* = 0.002) and isovalerate (*P* = 0.001) in AC at PND 32 compared with CONT pigs. By PND 61, no significance was observed in the concentrations of DM (*P* = 0.382), total VFA (*P* = 0.678), or any individual VFA on either absolute or relative bases in AC contents.

**Table 9:** Effect of iron status on volatile fatty acid concentrations in ascending colon contents^a^.

		**Diet**			
**Measure**	***N*^b^**	**CONT**	**ID**	**Pooled SEM**	***P*-value^c^**
**PND 32**					
Dry Matter, %	13	22.21	11.77	4.404	0.006
**Absolute concentration**, **μmol/g DM**					
Total VFA	13	349.67	859.51	183.390	0.003
Acetate	13	197.97	566.36	110.770	0.002
Propionate	13	88.81	171.76	43.692	0.018
Butyrate	13	36.77	80.21	19.862	0.012
Valerate	13	8.98	20.16	4.234	0.025
Isobutyrate	13	7.53	9.14	1.643	0.314
Isovalerate	13	8.90	10.96	2.083	0.321
**Relative profile, % of total VFA**					
Acetate	13	56.24	66.95	1.186	<0.001
Propionate	13	25.36	19.12	0.921	<0.001
Butyrate	13	10.21	9.02	0.653	0.213
Valerate	13	2.42	2.28	0.276	0.720
Isobutyrate	13	2.617	1.102	0.296	0.002
Isovalerate	13	2.93	1.33	0.245	0.001
**PND 61**					
Dry Matter, %	20	16.44	19.36	5.536	0.382
**Absolute concentration**, **μmol/g DM**					
Total VFA	20	647.34	610.93	197.330	0.678
Acetate	20	321.63	309.54	92.764	0.800
Propionate	20	194.44	163.66	61.291	0.363
Butyrate	20	105.31	109.83	39.239	0.753
Valerate	20	17.22	17.25	4.317	0.992
Isobutyrate	20	3.67	4.42	0.636	0.283
Isovalerate	20	5.77	6.24	0.730	0.585
**Relative profile, % of total VFA**					
Acetate	20	49.68	50.80	1.545	0.615
Propionate	20	29.27	26.09	1.664	0.194
Butyrate	20	16.56	18.01	1.191	0.316
Valerate	20	2.80	3.02	0.351	0.578
Isobutyrate	20	0.67	0.86	0.214	0.302
Isovalerate	20	1.05	1.23	0.347	0.497

a *Data presented as mean and pooled standard error of the means (SEM) for each dietary treatment group. Main effects of dietary treatment (Diet; CONT vs ID) are presented. CONT, control diet; ID, iron deficient diet; PND, postnatal day; SEM, standard error of the mean; DM, dry matter basis; VFA, volatile fatty acid*.

b *Total number of observations used*.

c *P-values for the main effect of early-life dietary iron status*.

#### Feces

In feces, DM was decreased (*P* < 0.001) and total VFA concentration was increased (*P* = 0.001) in feces from ID pigs compared with CONT pigs at PND 32 (Table 10). At the end of phase 1, concentrations of individual VFA on an absolute basis were increased in fecal samples of ID pigs, including acetate (*P* < 0.001), propionate (*P* = 0.001), and valerate (*P* = 0.007). Relative to the total VFA concentration, acetate was increased (*P* < 0.001) in ID pigs compared with CONT animals, and propionate (P = 0.001), butyrate (*P* < 0.001), isobutyrate (*P* < 0.001), and isovalerate (*P* < 0.001) were all decreased. At study conclusion, no effects of dietary early-life iron status were evident in feces, including concentrations of DM (*P* = 0.612), total VFA (*P* = 0.719), or any individual VFA on either absolute or relative bases.

**Table 10:** Effect of iron status on volatile fatty acid concentration in feces^a^.

		**Diet**			
**Measure**	***N*^b^**	**CONT**	**ID**	**Pooled SEM**	***P*-value^c^**
**PND 32**					
Dry Matter, %	23	53.54	26.05	2.905	<0.001
**Absolute concentration**, **μmol/g DM**					
Total VFA	23	143.14	302.80	96.985	0.001
Acetate	23	81.48	199.58	66.553	<0.001
Propionate	23	30.09	60.64	17.957	0.001
Butyrate	23	18.25	23.43	9.452	0.279
Valerate	23	2.78	5.20	1.158	0.007
Isobutyrate	23	4.11	6.18	1.173	0.060
Isovalerate	23	5.22	6.59	0.932	0.300
**Relative profile, % of total VFA**					
Acetate	23	47.65	63.12	1.540	<0.001
Propionate	23	23.97	21.06	0.551	0.001
Butyrate	23	14.37	7.27	0.971	<0.001
Valerate	23	2.68	2.22	0.232	0.165
Isobutyrate	23	4.47	2.62	0.440	<0.001
Isovalerate	23	6.08	2.95	0.882	<0.001
**PND 61**					
Dry Matter, %	20	23.64	22.63	1.762	0.612
**Absolute concentration**, **μmol/g DM**					
Total VFA	20	350.03	387.22	71.801	0.719
Acetate	20	187.55	207.61	35.380	0.694
Propionate	20	85.38	95.52	23.764	0.834
Butyrate	20	53.35	60.59	11.541	0.663
Valerate	20	9.68	11.47	2.555	0.627
Isobutyrate	20	5.98	6.44	0.689	0.643
Isovalerate	20	8.08	8.60	1.055	0.738
**Relative profile, % of total VFA**					
Acetate	20	54.62	55.01	1.388	0.823
Propionate	20	22.51	22.41	2.252	0.956
Butyrate	20	15.28	15.16	1.026	0.934
Valerate	20	2.64	2.74	0.177	0.685
Isobutyrate	20	2.19	1.98	0.320	0.649
Isovalerate	20	2.99	2.70	0.485	0.681

a *Data presented as mean and pooled standard error of the means (SEM) for each dietary treatment group. Main effects of dietary treatment (Diet; CONT vs ID) are presented. CONT, control diet; ID, iron deficient diet; PND, postnatal day; SEM, standard error of the mean; DM, dry matter basis*.

b *Total number of observations used*.

c *P-values for the main effect of early-life dietary iron status*.

## Discussion

### Microbiota

The diet provides nutrients for both the host and resident microbes within the lumen of the GIT (31), thus microbial composition of the GIT is heavily influenced by nutrition (32). Mounting evidence suggests that iron status has a significant impact on the microbiota by altering microbial diversity in the GIT and the growth of potentially-pathogenic, iron-requiring bacteria vs. non-iron-requiring bacteria (33–37). However, it should be noted that the majority of these studies were conducted to evaluate the effects of iron supplementation vs. deficiency. Few studies have characterized the effects of ID or IDA on the microbiota, and, to our knowledge, most studies that have characterized the effects of ID on the microbiota were performed in rodents (37, 38). As previously mentioned, the microbiota is known to have long-lasting effects on gastrointestinal, autoimmune, and metabolic diseases (5, 6). Given the prevalence of ID (1) and that early microbial colonization of the GIT can have lasting effects on the health and development of infants (5, 6, 15), understanding the effects ID has on the developing microbiota is imperative.

In this study, young pigs were provided an iron-adequate milk replacer, similar to human infant formula, or ID milk replacer through approximately 4 weeks of age followed by a period of dietary iron repletion. Sequencing of bacterial 16S rRNA genes was utilized to evaluate the effect of ID on the microbiome. We discovered through PCoA and PERMANOVA analyses that the microbiota of ID pigs differed significantly from that of CONT pigs in both AC and fecal samples at PND 32. In accordance with previous studies evaluating the microbiota of infants and pigs (8, 39–41), the main bacterial phyla in our study included Bacteroidetes, Firmicutes, and Proteobacteria. At the genera level, our findings indicate that the ID diet created an environment in the GIT in which non-iron-requiring, beneficial gut bacteria such as *Lactobacillus* and *Bifidobacterium* thrived.

The current study found that relative abundances of *Prevotella* and closely-related species, [*Prevotella*], were higher in both AC and feces of ID pigs. Our results are comparable with a previous report demonstrating that fecal samples of anemic Kenyan infants provided a non-iron-fortified micronutrient powder displayed increased abundance of *Prevotella* compared with infants who received an iron-fortified micronutrient powder (33), though it should be noted that this effect could be due to other confounding factors. Further, a study conducted in Sprague Dawley rats comparing the effects of varying iron supplementation methods, by providing an ID control diet in comparison to various methods of iron supplementation, also noted increased amounts of *Prevotella* in ID animals (38). However, Pereira et al. (38) observed a decrease in [*Prevotella*] in ID rats, which is contrary to our observations. This inconsistency could be related to the difference in species, or to the varying methods used to quantify the microbiota by Pereira and colleagues, which was noted as a limitation to their study (41).

The observed higher relative abundance of *Bifidobacterium* in feces and *Lactobacillus* in both AC and feces in ID pigs compared with CONT pigs in our study is congruent with other studies across multiple species (37, 42). It should be noted that most other researchers evaluated iron supplementation, and, thus, found that higher iron intake was related with decreased amounts of *Bifidobacterium* and *Lactobacillus* (16, 35, 43). This was expected, as these two genera are known to be among the few that are non- or low-iron requiring bacteria (44, 45). Interestingly, the iron-requiring bacteria *Escherichia/Shigella* (46, 47) were higher in feces of ID pigs compared with CONT pigs. Typically, potentially-pathogenic bacteria, such as *Escherichia*/*Shigella*, are decreased in low-iron environments. Further, it has been established that bacteria such as *Bifidobacterium* and *Lactobacillus* directly compete with *Escherichia* for resources (36, 37). Given that the observed prevalence of *Bifidobacterium* and *Lactobacillus* were higher in feces of ID pigs, the increase in the relative abundance of *Escherichia/Shigella* is even more surprising. To our knowledge, only one other study has reported an increase in *Escherichia* abundance in anemic animals (48), and it was related to the high prevalence of diarrhea. It has been accepted that some strains of *Escherichia/Shigella* are associated with diarrhea in infants (49). Fecal moisture content, expressed inversely as dry matter content, revealed that ID pigs had a 50% more moisture in feces compared with CONT pigs, thus it is possible that diarrhea was present, possibly associated with the higher levels of *Escherichia*/*Shigella* detected in ID pigs. It is also well-established that ID and IDA can lead to diminished immune function and higher susceptibility to pathogens (50, 51). Despite being iron-requiring bacteria, the greater susceptibility of ID pigs to illness may also contribute with the higher amount of *Escherichia/Shigella* observed. However, given that *Escherichia/Shigella* are opportunistic pathogens, the growth of these bacteria in ID pigs warrants future research to determine why they were increased so markedly in an ID environment.

Similar to *Escherichia* and *Shigella*, some members of *Bacteroides*, such as strains of *B. fragilis* are potentially-pathogenic, iron-requiring microbes, but they have also been recognized as having a beneficial relationship with the host when contained within the intestinal lumen (5, 52). *Bacteroides* is also known to be one of the most abundant genera found in both pigs (8, 41) and humans (53, 54). As such, *Bacteroides* represented a large proportion of the bacterial makeup of the AC contents of both CONT and ID pigs, but remained high only in the feces of CONT pigs, and was greatly decreased in feces of ID pigs. The difference in abundance of *Bacteroides* in the current study between AC and feces may be attributed to the amount of iron that is available between the two sites of the large intestine. *Bacteroides* is extremely proficient in its ability to scavenge for iron (55), which, although low in the proximal colon of the ID group, would have been comparatively more available than in the distal colon. Ultimately, it is unclear why the relative abundance of *Bacteroides* is only reduced in the feces of ID pigs compared with CONT pigs, but the subsequent decrease in *Bacteroides* in feces of ID animals, or its increase in cases of iron supplementation, has also been noted elsewhere (33, 37, 42, 56). No previous studies have evaluated the microbiota in AC, so future studies evaluating the regional abundances of *Bacteroides* in the context of iron status are warranted.

*Clostridium* from the families of *Clostridiaceae, Lachnospiraceae*, and *Ruminococcaceae* were higher in the feces of CONT pigs compared with ID pigs. The few studies assessing the effects of iron on the microbiota have focused on the *Clostridium* genera, however, one study noted an increase in various strains of *Clostridium* in infants given iron-supplemented micronutrient powders compared with infants who were not iron supplemented (33). Further, Wang and colleagues compared the microbiota of breastfed vs. formula-fed infants in the U.S. (5), and noted that levels of *Clostridium* were higher in formula-fed infants. It is well-established that human milk is low in iron (23, 57), and it is hypothesized that the iron in human milk is bound to lactoferrin to prevent use by potentially-pathogenic bacteria and heighten iron bioavailability to the infant (58). Further, infant formula in the U.S. is supplemented with iron at 12 mg/L (44, 59), and potentially-pathogenic members of the *Clostridium* genera are also iron-requiring bacteria (45). In the current study, CONT pigs were provided an iron-fortified milk replacer similar to human infant formula, whereas the ID pigs were provided a low iron formula with iron content similar to that in porcine and human milk. Taken together, these findings complement the higher relative abundances of various *Clostridium* strains in the CONT group vs. ID group.

After transitioning all pigs to an iron-replete diet, PCoA and PERMANOVA analyses at PND 61 showed that the microbiota of ID pigs no longer differed from that of CONT pigs in AC or feces. No significance was observed at the phylum level. At the genera level, the current study found that consumption of a common series of iron-adequate diets by both groups allowed for stabilization of the microbiota following early-life ID, with only three bacteria showing significance between ID and CONT pigs in AC and feces combined.

The observed decrease in the relative abundance of *Prevotella* in AC of ID pigs compared with CONT pigs at PND 61 was compelling given it was found to be increased at PND 32 in the ID group. It is unclear why this shift occurred, and future research should seek to elucidate the varying results seen in *Prevotella* in association with iron status across multiple studies (33, 38). *Bifidobacterium* remained higher only in AC of ID pigs compared with CONT pigs. Although the relative abundance of *Bifidobacterium* being increased in the ID group is congruent with findings at PND 32, it is interesting that *Bifidobacterium* was found to be higher in feces alone at PND 32 and in AC alone at PND 61. Further, given *Bifidobacterium* is among the few categorized as low-iron requiring bacteria (44, 45), the higher relative abundance after ID pigs were transitioned to an iron-replete diet was confounding, and these findings warrant future research.

It is important to highlight that even though the ID group showed lower concentrations of most potentially pathogenic bacteria and increased levels of beneficial gut bacteria at PND 32, the negative side effects known to be associated with IDA such as increased susceptibility to infection and disease (60), altered cognitive development (61, 62), and decreased growth performance (17) outweigh any potential benefits on the microbiota. Due to the high amount of genera significantly altered in the ID group at PND 32, we chose to speak mainly to those known to be effected by iron status. However, future research should be conducted to look into alterations in microbes not elucidated here. It should be noted that, although there was seemingly a full recovery of the microbiota of ID pigs similar to that of CONT pigs by PND 61, shifts from a liquid to a solid diet is known to strongly alter the microbial composition of the GIT, overshadowing other biological and environmental influences affecting microbial composition (63, 64). Further, the human infant's microbiota is thought to stabilize and become more “adult-like” after 1 year of age (65, 66). It is possible a similar effect could be seen in the pig, and this warrants further research, Overall, these were factors that could not be controlled for in the current study.

### Volatile Fatty Acid Composition

Volatile fatty acids are the primary end-products of microbial fermentation in the hindgut (67). Thus, the diet and the composition of the microbiota play significant roles in VFA formation (7, 68). At PND 32, we observed increases in total VFA concentrations in both AC and feces in ID pigs compared with CONT pigs. Given that CONT and ID milk replacers differed only in the level of iron, a non-fermentable nutrient, it can be surmised that the differences in VFA profiles between CONT and ID pigs stem directly from the shift in the microbiota due to early-life iron deficiency. A previous study comparing the microbial and VFA profiles of European and rural African children found that increased amounts of bacteria such as *Prevotella*, known for their fermentative capabilities of insoluble fibers, led to increased VFA concentrations (31). The ID pigs in this study displayed significantly higher relative abundances of *Prevotella* at PND 32 compared with CONT pigs, thus the higher levels of *Prevotella* and closely-related species could have contributed to the higher overall amount of VFA observed in the ID group. It has also been noted that VFA production via bacterial fermentation can lead to lower intestinal pH, subsequently increasing solubility of minerals such as iron and thereby improving their absorption (32, 69).

Further, Bouglé et al. performed a study utilizing the Ussing chamber to evaluate iron absorption throughout the GIT in Sprague-Dawley rats. They concluded that the ascending colon could be a noteworthy location for iron absorption (lower absorption rates than in the duodenum, which is the main site of iron absorption, but higher than more distal parts of the small intestine) (70). Bougle also noted that VFA, especially propionate, can enhance this effect (70). Interestingly, absolute amounts of propionate were higher at PND 32 in ID pigs compared with CONT pigs in both AC and feces in our study. Although we do not have direct measures of pH in AC and feces, we speculate that the higher amount of propionate and total VFA production observed in ID pigs could be a compensatory effect to counteract severe iron deficiency by allowing greater chances of any available iron to be absorbed. It should be noted that other studies evaluating VFA in the context of iron deficiency did not observe comparable effects to our study. Dostal et al. actually noted decreased amounts of VFA in a study evaluating the effects of iron deficiency and supplementation on the microbiota in Sprague-Dawley rats (37). However, Dostal et al. also found that VFA production between an ID and iron-sufficient group of rats were not significantly different from each other in a later study (34), though these variations could stem from the dissimilarities in model, as the rat practices coprophagy. Further, it should be noted that Dostal et al. reported VFA concentration as mmol/L without correcting for DM as done in the current study. Pigs in the current study are largely separated from their fecal material, making the results seen more representative than rodent studies. Taken together, the effects iron deficiency have on VFA profiles appears to vary across studies, which may require further investigation.

Following dietary iron repletion throughout phase 2 of the study, VFA profiles of ID pigs recovered to levels comparable to those observed in CONT pigs by PND 61. Recovery of VFA profiles of ID pigs to match that of CONT pigs was congruent with the finding that the microbial composition of ID pigs matched that of CONT pigs by PND 61. Taken together, these findings suggest that early-life iron deficiency has a significant effect on the microbiota, but recovery of the VFA and microbiota profiles are possible. Although it has been shown that a full recovery of the microbiota after a period of early-life iron deficiency is possible, future studies are necessary to establish precisely how long repletion of iron in the diet is necessary to see such effects.

## Conclusion

Herein, a severely anemic pig model was created through dietary manipulation alone. We observed altered VFA profiles at the end of phase 1, with ID pigs displaying increased VFA production in AC and feces. The microbiota was also significantly affected, and though greater differences were found in feces of ID pigs compared with CONT pigs, alterations in AC were elucidated in the separation of the two groups by PCoA and PERMANOVA analyses at PND 32. Apart from the finding that *Esherichia/Shigella* abundances were greater in ID pigs, there tended to be lower relative abundances of potentially pathogenic bacteria such as *Bacteroides* and *Clostridium* in ID pigs, and higher amounts of beneficial bacteria such as *Bifidobacterium* and *Lactobacillus* during phase 1.

Overall, these findings indicate that early-life iron deficiency affects the microbiota and, subsequently, VFA concentrations and profiles. Further, that iron is an essential nutrient for proper development of the neonatal microbiota. Volatile fatty acid concentration and profiles were recovered to levels comparable to those seen in CONT pigs at study conclusion. Evaluation of the microbiota at study conclusion also revealed no significant differences between ID and CONT pigs in PCoA and PERMANOVA analyses, suggesting that consumption of a common series of iron-adequate diets by both groups allowed for stabilization of the microbiota following early-life ID. However, future studies should seek to wholly characterize the influence of iron on the makeup of the microbiota after a time of dietary iron repletion without shifting from a liquid to a solid diet. This will allow microbial alterations due to the shift in diet matrix alone to be controlled for. The young pig proves to be an optimal translational model to study the effects of a micronutrient deficiency, specifically an iron deficiency, and how it will affect the development of the microbiota of both pigs and human infants. Further, it highlights a critical window during which adequate dietary iron intake is imperative to establish the microbiota. Lastly, future work should seek to further establish the duration of dietary iron repletion needed to fully reverse the effects of early-life IDA.

## Data Availability

The datasets generated for this study can be found in NCBI Sequence Read Archive (SRA), PRJNA543532.

## Ethics Statement

All animal and experimental procedures were in accordance with the National Research Council Guide for the Care and Use of Laboratory Animals and approved by the University of Illinois at Urbana-Champaign Institutional Animal Care and Use Committee.

## Author Contributions

All authors were involved in study design and implementation, data acquisition, analysis, and interpretation. LK wrote the manuscript. MW, SD, and RD read and approved the final version.

### Conflict of Interest Statement

The authors declare that the research was conducted in the absence of any commercial or financial relationships that could be construed as a potential conflict of interest.

## Abbreviations

AC: ascending colon content
CONT: control diet
GIT: gastrointestinal tract
ID: iron deficient diet
PCoA: principal co-ordinate analysis
PCR: polymerase chain reaction
PERMANOVA: permutational multivariate analysis of variance
PNCL: Piglet Nutrition and Cognition Laboratory
VFA: volatile fatty acids
VMRF: Veterinary Medicine Research Farm.
